# Alterations in bile acid metabolites associated with pathogenicity and IVIG resistance in Kawasaki disease

**DOI:** 10.3389/fcvm.2025.1549900

**Published:** 2025-02-20

**Authors:** Xinqi Wang, Linli Han, Jiyang Jiang, Zhenxin Fan, Yimin Hua, Libang He, Yifei Li

**Affiliations:** ^1^ State Key Laboratory of Oral Diseases & National Clinical Research Center for Oral Diseases & Department of Cariology and Endodontics, West China Hospital of Stomatology, Sichuan University, Chengdu, China; ^2^ Key Laboratory of Bioresources and Eco-environment (Ministry of Education), College of Life Sciences, Sichuan University, Chengdu, Sichuan, China; ^3^ Key Laboratory of Birth Defects and Related Diseases of Women and Children of MOE, Department of Pediatrics, West China Second University Hospital, Sichuan University, Chengdu, Sichuan, China

**Keywords:** Kaeasaki diseases, metabolome analysis, coronary artery injuries, IVIG resistance, bile acid, lipids

## Abstract

**Background:**

Kawasaki disease (KD) primarily affects children as an acute systemic vasculitis. Numerous studies indicated an elevated risk of cardiovascular disease due to metabolic disturbances. Despite this knowledge, the specific metabolic modes involved in KD remain unclear.

**Methods:**

We examined the metabolome of individuals with 108 KD and 52 non-KD controls (KD vs. nKD) by ultraperformance liquid chromatography (UPLC) and tandem mass spectrometry (MS).

**Results:**

Differential analysis uncovered the disturbed production of bile acids and lipids in KD. Furthermore, we investigated the impact of treatment, intravenous immunoglobulin (IVIG) resistance, and coronary artery (CA) occurrence on the metabolome. Our findings suggested that IVIG treatment alters the lipid and amino acid metabolism of KD patients. By orthogonal projections to latent structures discriminant analysis (OPLS-DA), there was no significant difference between the coronary injury groups and non-coronary injury groups, and IVIG resistance didn't appear to cause the metabolic change in KD patients.

**Conclusions:**

Patients with KD exhibit metabolic abnormalities, particularly in bile acids and lipids. IVIG interventions may partially ameliorate these lipid abnormalities.

## Introduction

Kawasaki disease (KD) is a multisystem inflammatory condition predominantly affecting children under five years of age, characterized by distinctive clinical manifestations including persistent fever, oral mucosa changes, hyperemic bilateral conjunctiva, extremity changes, and cervical lymphadenopathy (1–3). This condition significantly impacts both physical and mental health, necessitating prompt diagnosis and intervention (3). Intravenous immunoglobulin (IVIG) remains the primary therapeutic approach, functioning through multiple mechanisms including modulation of inflammatory cytokine expression, reduction of toxicity, improvement of vascular endothelial function, and mitigation of coronary artery lesions (CAL) (4). However, IVIG resistance poses a significant clinical challenge, as affected patients may develop serious complications, particularly CAL. Current biochemical indicators associated with KD, such as erythrocyte sedimentation rate, C-reactive protein, and procalcitonin, lack disease specificity, highlighting the urgent need for developing specific diagnostic biomarkers to enable more effective clinical management of KD (3, 5–10).

Metabolomics offers a comprehensive approach to understanding systemic metabolic changes, providing both quantitative and qualitative methods for extensive biomarker detection and precise disease state classification. This analytical approach has proven particularly valuable in identifying cardiovascular disease biomarkers. Studies have revealed specific metabolic signatures associated with various cardiovascular conditions: valine has been identified as a protective factor in acute myocardial infarction, while elevated serum creatinine levels indicate an increased risk (11). Furthermore, several metabolites, including medium-chain acylcarnitines, short-chain and long-chain dicarboxylacylcarnitines, branched-chain amino acids, and fatty acids, have demonstrated independent predictive value for future cardiovascular events (12). In the context of cerebral infarction, key metabolic biomarkers have been identified, including folic acid, cysteine, S-adenosyl homocysteine, and oxidized glutathione (13).

Research has established that KD is characterized by significant metabolic dysregulation. Early studies identified urinary neopterin as a predictive biomarker for coronary artery abnormalities, while subsequent research revealed that low serum 25(OH)-vitamin D levels, crucial for immunological regulation, may contribute to coronary artery complications (14). The growing recognition of metabolites’ role in KD pathogenesis has led to increased application of metabolomics in recent research (15). Notably, lipidomics investigations have yielded important insights: Japanese researchers demonstrated elevated oxidized phosphatidylcholine (PC) levels during KD's acute phase, while another study focusing on IVIG-resistant KD patients identified significant pre- and post-treatment variations in lysophosphatidylcholine (LPC) and lysophosphatidylethanolamine (LPE) (16, 17). Although these studies have established the relevance of metabolic investigations in KD, comprehensive understanding of KD metabolism remains limited. The field has also benefited from other omics approaches, including genomics and metagenomics, which have provided valuable insights from genetic and microbial perspectives, highlighting the crucial role of multi-omics approaches in advancing KD research (18–20).

In this study, we conducted untargeted metabolomic profiling to characterize the metabolic signatures distinguishing KD patients from non-KD controls. Our comprehensive analysis encompassed 108 KD patients and 52 non-KD controls, examining not only the baseline metabolic differences but also investigating how these profiles were influenced by medical intervention, IVIG resistance, and the development of CAL.

## Results

### Blood serum samples collection and untargeted metabolome assessments

To establish the profiles of the metabolic characteristics of KD patients, blood serum samples from 108 patients with KD and 52 non-KD controls were collected (KD vs. nKD) for untargeted metabolome assessments. Initially, the serum samples had been collected before or after IVIG administration in KD patients, which were used to compare the differences between metabolites pre-treatment and post-treatment. Then, comparisons were also made based on whether the patients underwent resistance in IVIG initial supplementation (IVIG vs. rIVIG), and whether coronary artery lesions (CAL) had been observed in the acute or sub-acute term when KD onset (CAL vs. nCAL). Moreover, in order to further exploration of the potential molecular mechanisms of the metabolic substrates in the pathogenicity and pathophysiological process of KD, the patients who were diagnosed with KD had been recognized by various clinical manifestations (Supplementary Tables S1–S4). Essentially, a total of 1,069 metabolites were detected between KD and nKD (Supplementary Table S5), including lipids, organic acids, organic heterocyclic metabolites, and others.

### Alternation of metabolism had been identified in KD patients

**Figure 1:**
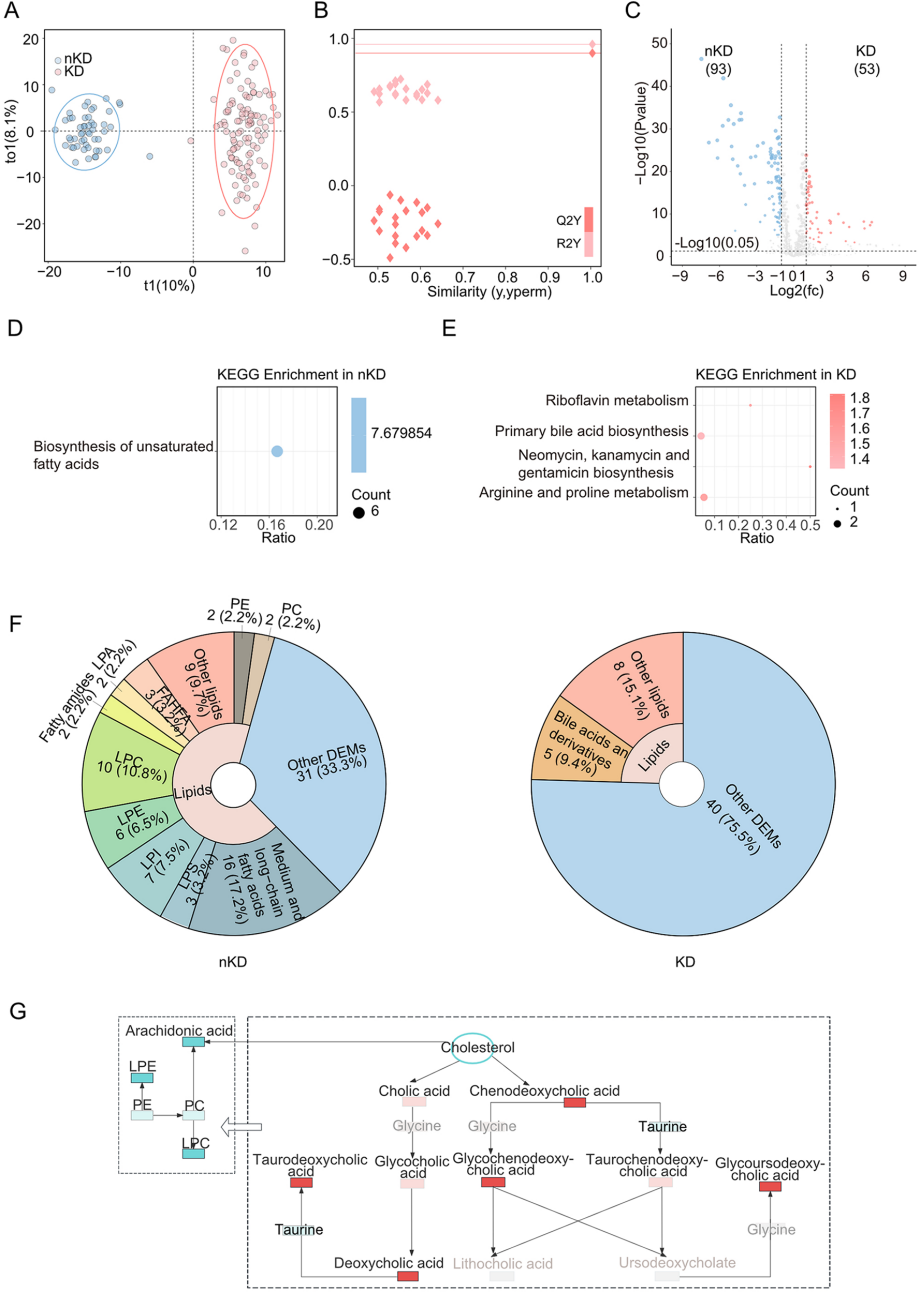
Metabolomics between KD and nKD. **(A)** OPLS-DA scores scatter plot of KD and nKD. **(B)** OPLS-DA permutation test. The R2X, R2Y, and Q2Y were 0.229, 0.96, and 0.9. **(C)** Volcano plot for the OPLS-DA model. Blue indicated upregulated O_DEMs in nKD and red indicated upregulated O_DEMs in KD. Only points with VIP greater than 1 have a color in the plot. **(D)** KEGG pathway enrichments of nKD **(E)** KEGG pathway enrichments of KD. **(F)** The proportions of lipids in nKD(left) and KD(right)'s DEMs pie chart. **(G)** Schema of dysregulated bile acid metabolism. Brightening color boxes represent metabolites that had been identified and have significant differences; darkening color boxes represent metabolites that had been identified but did not have significant differences; grey boxes represent metabolites that had not been identified; red showed a positive correlation and blue showed a negative correlation; PE, Phosphatidylethanolamine.

Firstly, differential analyses were performed to detect the differentially expressed metabolites among various groups. Moreover, to achieve more scientific and convincing results, orthogonal projections to latent structures discriminant analysis (OPLS-DA) were involved. The identified metabolites were clustered based on OPLS-DA, and the OPLS-DA score plots were visualized with the first principal component (t1, 10%) and the orthogonal component (to1, 8.1%) (Figure 1A), presenting two separated clusters between KD and nKD samples which indicated a significant difference in metabolic substrates analysis. Moreover, further validation plots were obtained through 200 permutation tests (Figure 1B). The R2Y and Q2Y scores were 0.96 and 0.9, which demonstrated the OPLS-DA as a satisfied analytical model with test effectiveness. 146 differentially expressed metabolites (O_DEMs) (VIP value >1, *P*-value <0.05, and |Log2FC|>1) were targeted by OPLS-DA method (Figure 1C). Next, we involved KEGG enrichment for O_DEMs (Figures 1D,E). It demonstrated that the down-regulated O_DEMs in KD enriched in the biosynthesis of unsaturated fatty acids pathway. The O_DEMs demonstrated significant enrichments in lipids, mainly in fatty acid metabolism, which indicated the alternations of fatty acid metabolism contributed to the pathogenicity of KD.

Then all the O_DEMs had been enrolled for the next analysis. Interestingly, lipids-related metabolites took a major proportion (66.7%) of nKD's O_DEMs (Figure 1F, left). Moreover, multiple lipids-related metabolites found among all the DEMs were associated with hepatic function. The lipid metabolites, such as bilirubin, uric acid were identified as both reduced in KD patients (Supplementary Table S5). Notably, the regulation of bile acids were found to be elevated in KD patients with significant enrichments of primary bile acid biosynthesis (Figure 1E), which were critical in fatty acid metabolism (21). To further analysis the specific metabolites had been identified to participate in what metabolic processes, such as deoxycholic acid. We linked the bile acid and lipid metabolism pathways to illustrate the alternations of fatty acid metabolisms involved in KD, which were induced by the down-regulated cholesterol and upregulated bile acids resulting in the decreased biosynthesis on fatty acid (Figure 1G).

### IVIG alleviated the dysfunction in fatty acid metabolism in KD patients

**Figure 2:**
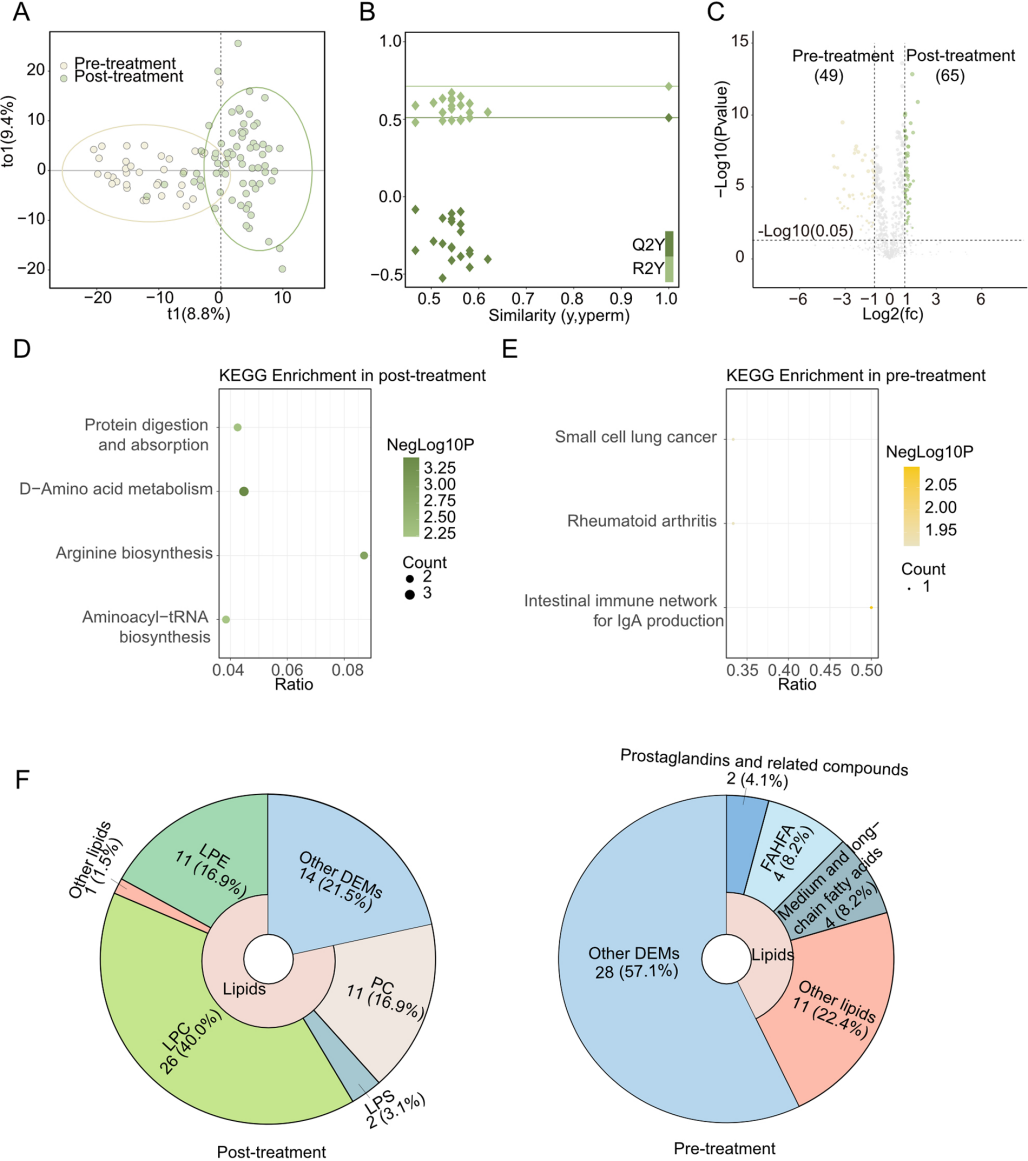
Metabolomics between post-treatment and Pre-treatment. **(A)** OPLS-DA scores scatter plot of Post-treatment and Pre-treatment. **(B)** OPLS-DA permutation test. The R2X, R2Y, and Q2Y were 0.182, 0.713, and 0.51. **(C)** Volcano plot for the OPLS-DA model. Yellow indicated upregulated O_DEMs in Pre-treatment and green indicated upregulated O_DEMs in Post-treatment. Only points with VIP greater than 1 have a color in the plot. **(D)** KEGG pathway enrichments of Post-treatment **(E)** KEGG pathway enrichments of Pre-treatment. **(F)** The proportions of lipids in Post-treatment (left) and Pre-treatment (right)'s DEMs pie chart.

Next, we focused on the effects of the medical treatment on KD's metabolism, so we performed an OPLS-DA analysis. The OPLS-DA score plot is shown in Figure 2A, with 8.8% t1 and 9.4% to1, confirming a clear separation of results between post-treatment and pre-treatment groups. The 200 permutation tests were used to determine the OPLS-DA models’ reliability, showing the OPLS-DA model was reliable (Figure 2B). And OPLS-DA detected 114 O_DEMs (VIP value >1, *P*-value <0.05, and |Log2FC|>1) (Figure 2C).

Up-regulated O_DEMs in Post-treatment were enriched in aminoacyl-tRNA biosynthesis, protein digestion and absorption, arginine biosynthesis and D-amino acid metabolism KEGG pathway (Figure 2D). Meanwhile, down-regulated O_DEMs had different KEGG enrichment results, such as small cell lung cancer, et al. (Figure 2E). Notably, multiple lipids (LPE, etc.) (Figure 2F, left) and amino acids (DL-arginine, etc.) elevated in Post-treatment.

Comparing the CAL and nCAL, the difference is too rare to build a valid statistical model in OPLS-DA. In addition, the OPLS-DA method also didn't show any significant differences between IVIG and rIVIG.

### Weighted gene co-expression network analysis

**Figure 3:**
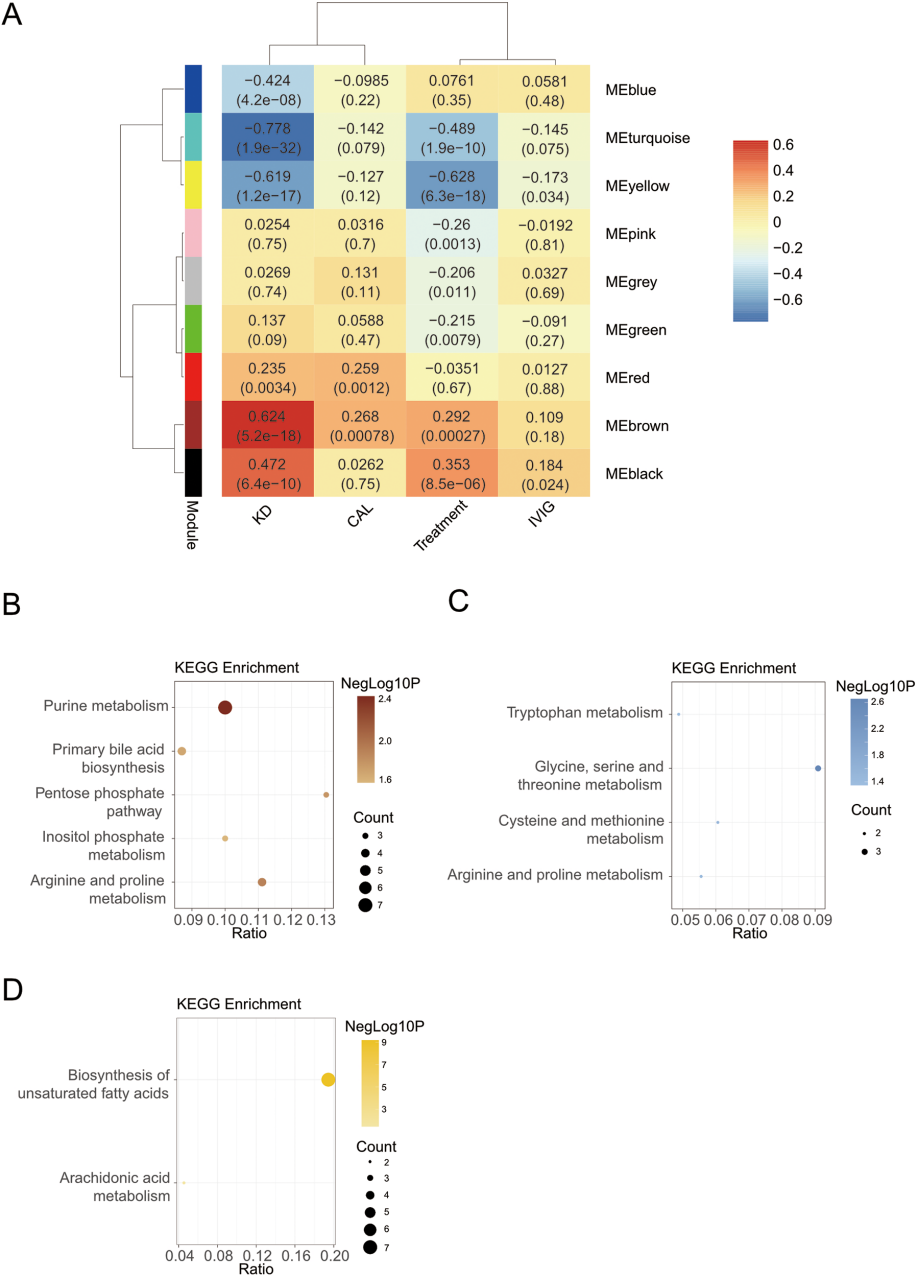
Screening markers for KD. **(A)** Module-trait relationships between WGCNA modules and KD. **(B)** The KEGG enrichment analysis of brown module. **(C)** The KEGG enrichment analysis of blue module. **(D)** The KEGG enrichment analysis of yellow module.

Weighted gene co-expression network analysis (WGCNA) was used to identify potential biomarkers and further grouped metabolites into 8 different modules (excluding the grey color module, Figure 3A). Five modules (including turquoise, yellow, brown, black and blue) were significantly related to KD factor (|r|≥0.4, *P*-value < 0.05), and two modules (including turquoise and yellow) were significantly related to the treatment factor. To characterize module functions, the metabolites of modules were used to perform KEGG database searches and literature-based functional mining. Surprisingly, some WGCNA modules merited attention. For example, both the turquoise and black modules were related to the bile acid metabolism (Supplementary Table S5) but showed opposite correlations in KD and after the IVIG treatment (Figure 3A). The turquoise module contained mainly non-bile acid metabolites related to bile acid metabolism, such as uric acid, phosphatidyl ethanolamine (PE), etc. While the black module contained mainly free bile acids, such as deoxycholic acid.

Next, we focused on modules with KEGG pathway enrichment results. Another module that was also related to bile acid metabolism was the brown module. The brown module had the strongest positive relation with KD (*r* = 0.624, *P*-value < 0.001), including metabolites involved in purine metabolism, bile acid metabolism, etc. This work was similar with the differential results by OPLS-DA analysis. Metabolites in the brown module were enriched in purine metabolism, primary bile acid biosynthesis, arginine and proline metabolism, pentose phosphate pathway, and inositol phosphate metabolism KEGG pathway (Figure 3B).

The blue module positively correlated only with the KD trait and contained metabolites mainly enriched in amino acid-related pathways (Figure 3C), such as glycine, serine and threonine metabolism. The blue module was not associated with the treatment trait. However, in the differential results, we found that multiple amino acids were down-regulated in the Post-treatment group. It may because that WGCNA places more emphasis on correlation and difference analysis focuses more on the difference.

The yellow module was also highly negatively correlated with KD and treatment traits and acted as the strongest correlation with treatment traits. The yellow module contained metabolites mainly enriched in the biosynthesis of unsaturated fatty acids and arachidonic acid metabolism (Figure 3D).

Similar to the differential results, there were no significant correlations between these modules and CAL and IVIG traits.

## Discussion

KD is an acute systemic vasculitis that primarily affects the heart and coronary arteries, while also impacting multiple visceral organs, including the liver—the key organ for bile acid synthesis and secretion. Our study revealed significant alterations in bile acid metabolism associated with KD.

The liver is the primary site for high-density lipoprotein (HDL) production. Once secreted, HDL binds with cholesterol to form HDL-cholesterol (HDL-C), which facilitates cholesterol transport back to the liver. HDL-C plays crucial roles in both vascular inflammation and calcification (22–26). In hepatic metabolism, cholesterol catabolism leads to the synthesis of primary bile acids. These primary bile acids undergo transformation by intestinal microbiota to form secondary bile acids, which are either excreted or reabsorbed through enterohepatic circulation (27–29). Both primary and secondary bile acids can undergo conjugation with glycine or taurine to form conjugated bile acids (30). Through Weighted Gene Co-expression Network Analysis (WGCNA), we observed distinct expression patterns: conjugated bile acids clustered in the brown module, while free bile acids were grouped in the black module.

Bile acids serve crucial physiological functions but can also act as double-edged swords in human health. While essential for normal metabolism, elevated bile acid levels can promote inflammation, and their chronic accumulation may result in cholestasis, leading to liver inflammation and injury. Although cholestasis has been occasionally observed in clinical cases of KD, none of our study samples exhibited this condition. This absence of cholestasis might be attributed to either well-controlled disease states in our samples or that the disease had not progressed to a more severe stage.

Our analysis of differentially expressed metabolites (DEMs) in KD revealed two significant changes: a decrease in cholesterol derivatives, such as 22(S)-Hydroxycholesterol, and an elevation in bile acid levels. Clinical laboratory findings (Supplementary Table S1) further demonstrated significant reductions in TC and HDL-C, while LDL-C remained unchanged. The observed decrease in TC and HDL-C likely stems from two mechanisms: reduced hepatic HDL production and enhanced cholesterol catabolism. This increased cholesterol breakdown subsequently led to elevated bile acid synthesis (31). Our results indicated disturbed bile acid metabolism in KD patients, suggesting compromised hepatic scavenging capacity resulting in bile acid accumulation. The liver-cardiovascular disease connection is well-documented in the literature, and our findings revealed multiple indicators of impaired hepatic clearance capacity, including abnormalities in liver metabolites, elevated liver function markers (ALT and LDH), and decreased bilirubin levels. This was further supported by reduced TB and DBIL in clinical tests.

Bile acids play essential roles in lipid metabolism by breaking down lipids into smaller molecules and facilitating their dispersion into oil-in-water colloidal particles. This process enhances lipid solubility in aqueous solutions, creating optimal conditions for lipid particle interactions and lipase activity, thereby promoting efficient lipid digestion and absorption. In KD, we observed widespread downregulation of multiple lipids, particularly those affecting vasoactive endothelial function, including lysophosphatidylcholine (LPC), lysophosphatidylethanolamine (LPE), and lysophosphatidic acid (LPA) (32–34). LPC and LPE serve dual roles as metabolites of phosphatidylcholine (PC) and phosphatidylethanolamine (PE) respectively, while also functioning as structural components of mammalian cell membranes. Studies have revealed diverse immunological effects of LPCs: saturated and monounsaturated forms exhibit pro-inflammatory properties, while polyunsaturated variants demonstrate anti-inflammatory effects (35, 36). Similarly, LPE has been shown to exert anti-inflammatory effects on macrophages and can potentially trigger protective immunity through natural killer T cell-dependent mechanisms (37, 38). Notably, all identified LPCs and LPEs were found among the downregulated overlapping differentially expressed metabolites (O_DEMs) in KD, with the majority clustering within the blue module.

Previous lipidomics studies in KD have demonstrated that oxidized phospholipids, particularly PCs, can trigger inflammatory signals leading to coronary arteritis (16). Our findings revealed that elevated bile acid levels were associated with decreased lipid concentrations, with these down-regulated lipids subsequently affecting vasoactive endothelial function. Furthermore, disturbances in bile acid metabolism could directly impact cardiovascular health, as bile acids can impair cardiac mitochondrial function, potentially leading to cardiomyopathy (39).

Comparing metabolite profiles before and after medical treatment revealed significant changes in both lipid and amino acid metabolism. Post-treatment analyses showed marked increases in various lipids, such as LPC, and amino acids, including citrulline. These changes are particularly significant as amino acids not only serve as protein building blocks but also promote endothelial cell proliferation and angiogenesis, while abnormal lipid metabolism can trigger vascular inflammation through immune cell activation, particularly macrophages (40). The therapeutic intervention effectively ameliorated these lipid disorders in KD patients, addressing the metabolic disturbances we previously described (41).

Our study involved several limitations. For example, even if our conclusions fitted the clinical laboratory examination, but still need more patients to validate conclusions. Our IVIG vs. rIVIG was inconclusive, probably because there were too few individuals in the rIVIG group due to sampling limitations, resulting in our failure to analyze metabolic difference results. And some content of research needed to be explored in depth. Our study comprehensively profiled the changes in metabolites related to KD, yet did not delve deeply into the pathogenesis of KD. Subsequent studies could use targeted metabolomics for more precise quantitative analysis, delving into the specific roles of metabolites in the pathogenesis of KD. For bile acid metabolism, many studies have shown that microbes in the body regulate it. A disorder in bile acid metabolism was found in our results, which may be related to the patient's microbial metabolism. There have been multiple microbial studies demonstrating interactions between bile acids and microbial populations (28, 42, 43). As for microbial studies in KD, the spotlight was on variations in microbial content. For example, Kinumaki et al. (44) revealed an elevated presence of Streptococcus spp. in the gut microbiota of patients in the acute phase of KD. So continued research combining metabolome and metagenomic may explore new biological pathways. We only used blood as the research object, and later studies can add substances such as urine and tissue fluid. We can also improve our study by determining the amount of undetected or non-significantly different substances in our study through methods such as targeting metabolome.

In summary, metabolomics analysis identified potential metabolic pathways in the KD. Our analyses suggested that significant changes in bile acid and lipid metabolism correspond to KD.

## Methods

### Study design and subjects

This study was approved by the Ethics Committee of West China Second University Hospital of Sichuan University (NO. 2020-092). Written informed consents were obtained from all subjects. Besides, another serum was collected and used later to determine the measurement of liver function, renal function, lipid level, and so on. The 108 participants involved in this research were enrolled from the West China Second University Hospital of Sichuan University from August 2022 to June 2023. The enrolled 52 controls were age-appropriate and sex-matched healthy children who were totally absent from the history of KD. A total of 108 consecutive KD children (57 males/51 females, average aged 3.19 ± 2.38 years) and 52 volunteer controls (21 males/31 females, average aged 3.99 ± 2.23 years) were included for blood samples collection and 160 in total.

### Inclusion and exclusion criteria

We recruited candidates for further analysis using the following inclusion criteria: (1) All patients should meet the diagnostic criteria for complete or incomplete diagnosis of KD as recommended by the AHA (2017), and the diagnosis should be confirmed by two physicians; (2) Echocardiography found coronary aneurysms in the acute or subacute phase; (3) Procedure questionnaire, basic information, clinical manifestations, hematological examination results, treatment procedures, echocardiogram results were collected; (5) In order to easy balance the bias from high-risk ages, the age of the included patients ranged from 1 to 10 years old, which was convenient to balance the bias of high-risk age; (6) Neither transthoracic echocardiography nor transcatheter angiography evaluated coronary features. Exclusion criteria included: (1) Patients with cardiovascular malformations; (2) The patient had been diagnosed with autoimmune disease before the onset of KD; (3) The patients had received anticoagulant or antiplatelet drugs before the onset of KD; (4) Patients who have undergone heart surgery; (5) Suspected myocarditis before KD; (6) Glucocorticoids were provided before IVIG; (7) Patients provided monoclonal antibodies, including tumor necrosis factor (TNF)-*α* or interleukin-6 antibodies; (8) Kawasaki disease diagnosed with macrophage activation syndrome or hemophagocytic lymphohistiocytosis; (9) No echocardiography was available to record KD in the acute and subacute phases.

### Ultra-performance liquid chromatography-tandem-mass spectrometry

The collected blood samples were kept in sodium heparin anticoagulation tubes. After centrifuging the blood samples in the field and dispensing their upper liquid layer, all samples were transferred to the laboratory in dry ice and kept in a −80°C cryogenic refrigerator until extraction was initiated. Serum samples were subjected to ultra-performance liquid chromatography-tandem-mass Spectrometry (UPLC-MS/MS) analysis. A HypersilGoldcolumn (C18) was used, with 5 mM ammonium acetate (A, for negative mode), 0.1% formic acid (A, for positive mode), and methanol (B). The mass spectrometer was operated in both positive and negative electrospray ionization (ESI+/ESI−) mode. The UPLC-MS/MS raw data were analyzed by the Metabolite Discoverer 3.3 (CD3.3, ThermoFisher), and raw data was extracted, peak-identified and QC processed. The qualitative and quantitative analysis of metabolites by matching peaks with the mzCloud, mzVault, and MassList databases. Then Metabolites were annotated using LipidMaps (http://www.lipidmaps.org) (45), HMDB (http://www.hmdb.ca/) (46), and KEGG (http://www.genome.jp/kegg) (47) databases.

### Metabolomics data were analyzed by statistical analysis

Differential metabolites analysis was conducted using the R package MetaboAnalystR4.0 (48). Preprocessing data with “Normalization(mSet, “MedianNorm”, “NULL”, “AutoNorm”, ratio = FALSE, ratioNum = 20)”. Processed data were subjected to statistical analyses to identify between-group DEMs. *P*-values are from hypergeometric tests. The part of OPLS-DA analysis used package ropls (49) function“opls” to get the variable important in projection (VIP) values of each metabolite. OPLS-DA scores scatter plot and OPLS-DA permutation test were also used package ropls. The screening criteria for O_DEMs were VIP value >1, *P*-value <0.05, and |Log2FC|>1. The KEGG database was used for pathway enrichment analysis to find enriched metabolic signaling pathways involving differential metabolites between two groups. The number of all human KEGG pathway metabolites equaled “N” (*N* = m + n) and the number of individual pathway metabolites equaled “m”. Names of DEMs were all converted to KEGG IDs starting with “C” followed by a five-digit number, and the number of DEMs enriched in the pathway was counted as “x” (q = x-1). For KEGG pathway enrichment analysis, enter the total number of metabolites as “k”. The *P*-value of each pathway was calculated using the “phyper” function that comes with the R language, and the pathways were finally screened for significance according to the *P*-value <0.05. Volcano plots and KEGG pathway annotations and enrichments plots were drawn using the ggplot2 package, and the Venn plot used an online tool (http://bioinformatics.psb.ugent.be/webtools/Venn/).

### Weighted gene co-expression network and receiver operating characteristic analyses

WGCNA was performed in R using the WGCNA package (50). Setting the soft power threshold at 8 to arrive at the network adjacency and a minimum module size of 30. The grey module contains all analytes that were not assigned to any of the other modules, and a total of 8 non-gray modules were generated. For the brown module, the correlation between every metabolite was calculated.

## Data Availability

The datasets presented in this study can be found in online repositories. The names of the repository/repositories and accession number(s) can be found in the article/Supplementary Material.

## Supplementary material

Supplementary Table S1 Clinical information of samples of KD and non-KD.

Supplementary Table S2 Clinical information of samples of Pre-treatment and Post-treatment.

Supplementary Table S3 Clinical information of samples of CAL and nCAL.

Supplementary Table S4 Clinical information of samples of IVIG and rIVIG.

Supplementary Table S5 Metabolites detected in the untargeted metabolomes of the 160 samples.
