# Supplementary material for: Alterations in bile acid metabolites associated with pathogenicity and IVIG resistance in Kawasaki disease
Source: Front Cardiovasc Med. 2025 Feb 20;12:1549900. doi: 10.3389/fcvm.2025.1549900 (PMC11882569; doi:10.3389/fcvm.2025.1549900)
Supplement: Supplementary Table S1 — Clinical information of samples of KD and non-KD. [file Table1.docx]

Table 1. Clinical information of samples of KD and non-KD

| Variables | KD (n=105(108#)) | Non-KD (n=52) | Significance |
| --- | --- | --- | --- |
| Age (years) | 3.19±2.38 | 3.99±2.23 | NS |
| BMI (kg/m^2^) | 16.62±2.44 | 15.35±1.63 | ^***^ |
| **Gender** |  |  | NS |
| Male | 54 (51%) | 21 (40%) |  |
| Female | 51 (49%) | 31 (60%) |  |
| **Ethnic** |  |  | ^***^ |
| Minorities | 4 (4%) | 7 (13.5%) |  |
| Han Nationality | 101 (96%) | 45 (86.5%) |  |
| WBC (×10^9^/L) | 14.45±6.16 | 8.75±2.9 | ^***^ |
| N (%) | 67.85±15.98 | 38.41±13.79 | ^***^ |
| L (%) | 23.24±13.43 | 53.28±13.75 | ^***^ |
| M (%) | 6.19±2.97 | 5.76±1.72 | NS |
| RBC (×10^12^/L) | 4.2±0.51 | 4.47±0.45 | ^**^ |
| HGB (g/L) | 110.47±11.15 | 119±9.64 | ^***^ |
| PLT (×10^9^/L) | 352.38±121.85 | 348.59±90.15 | NS |
| HCT (%) | 33.57±3.28 | 36.22±2.79 | ^***^ |
| PCT (%) | 0.34±0.11 | 0.34±0.08 | NS |
| CRP (mg/L) | 73.07±46.13 | 0.62±0.67 | ^***^ |
| ALT (U/L) | 61.42±75.2 | 17.51±7.43 | ^***^ |
| AST (U/L) | 46.96±44.47 | 37.9±11.24 | NS |
| AST/ALT | 1.37±0.82 | 2.34±0.73 | ^***^ |
| TB (mmol/L) | 10.91±13.63 | 6.47±2.72 | ^*^ |
| DBIL (mmol/L) | 5.9±10.94 | 2.09±1.23 | ^*^ |
| IDIL (mmol/L) | 4.94±3.37 | 4.5±1.77 | NS |
| ALB (g/L) | 40.55±4.62 | 44.96±2.95 | ^***^ |
| GLB (g/L) | 21.99±4.29 | 21.46±3.42 | NS |
| γGT (U/L) | 64.44±79.04 | 15.55±11.16 | ^***^ |
| LDH (U/L) | 314.2±89.44 | 277.75±52.82 | ^**^ |
| PA (mg/L) | 59.02±34.71 | 194.86±38.19 | ^***^ |
| ALP(U/L) | 198.81±63.68 | 249.63±57.02 | ^***^ |
| UN (mmol/L) | 3.3±1.1 | 5.22±1.46 | ^***^ |
| Cr (umol/L) | 26.73±6.35 | 27.76±8.25 | NS |
| CYSC (mg/L) | 0.81±0.17 | 0.82±0.12 | NS |
| UA (umol/L) | 209.61±72.65 | 264.47±72.64 | ^***^ |
| TC (mmol/L) | 3.3±0.71 | 3.87±0.87 | ^**^ |
| HDLC (mmol/L) | 0.75±0.34 | 1.33±0.26 | ^***^ |
| LDLC (mmol/L) | 2.93±3.06 | 2.36±0.97 | NS |

# We collected 108 KD individuals for metabolomic analysis, but the information was missing for 3, and the table is only for 105 individuals.

*<0.05; **<0.01;***<0.001;NS, not significant; BMI, body mass index; N, neutrophil; L, lymphocyte; M, monocyte; RBC, red blood cell; HGB, hemoglobin; PLT, platelet; HCT, Hematocrit; PCT, procalcitonin; CRP, C-reactive protein; γGT, γ glutamyltransferase; PA, serum prealbumin; ALP, alkaline phosphatase; CYSC, Cystatin C.

Continues data were presented as mean ± SD; categorical variables were presented as percentage.
