# Supplementary material for: Alterations in bile acid metabolites associated with pathogenicity and IVIG resistance in Kawasaki disease
Source: Front Cardiovasc Med. 2025 Feb 20;12:1549900. doi: 10.3389/fcvm.2025.1549900 (PMC11882569; doi:10.3389/fcvm.2025.1549900)
Supplement: Supplementary Table S2 — Clinical information of samples of Pre-treatment and Post-treatment. [file Table2.docx]

Table 2. Clinical information of samples of Pre-treatment and Post-treatment

| Variables | Pre-treatment (n=36) | Post-treatment (n=60) | Significance |
| --- | --- | --- | --- |
| Age (years) | 3.21±2.71 | 3.17±2.21 | NS |
| BMI (kg/m^2^) | 17.15±2.81 | 16.34±2.19 | NS |
| **Gender** |  |  | NS |
| Male | 19 | 35 |  |
| Female | 17 | 34 |  |
| **Ethnic** |  |  | NS |
| Minorities | 2 | 2 |  |
| Han Nationality | 34 | 67 |  |
| WBC (×10^9^/L) | 14.03±5.71 | 14.67±6.4 | NS |
| N (%) | 66.82±16.58 | 68.38±15.76 | NS |
| L (%) | 24.36±14.5 | 22.66±12.92 | NS |
| M (%) | 6.26±2.67 | 6.16±3.13 | NS |
| RBC (×10^12^/L) | 4.22±0.5 | 4.19±0.52 | NS |
| HGB (g/L) | 110.29±9.52 | 110.55±11.96 | NS |
| PLT (×10^9^/L) | 348.65±110.97 | 354.27±127.78 | NS |
| HCT (%) | 33.61±3.14 | 33.55±3.37 | NS |
| PCT (%) | 0.33±0.1 | 0.34±0.11 | NS |
| CRP (mg/L) | 66.19±41.02 | 76.51±48.41 | NS |
| ALT (U/L) | 73.31±82.82 | 55.21±70.75 | NS |
| AST (U/L) | 47.77±36.58 | 46.54±48.33 | NS |
| AST/ALT | 1.22±0.82 | 1.45±0.81 | NS |
| TB (mmol/L) | 12.47±15.53 | 10.09±12.55 | NS |
| DBIL (mmol/L) | 7.33±12.86 | 5.15±9.82 | NS |
| IDIL (mmol/L) | 5.14±3.03 | 4.84±3.55 | NS |
| ALB (g/L) | 40.73±4.74 | 40.45±4.59 | NS |
| GLB (g/L) | 21.55±3.57 | 22.23±4.64 | NS |
| γGT (U/L) | 80.37±82.45 | 56.12±76.51 | NS |
| LDH (U/L) | 309.51±73.98 | 316.64±96.97 | NS |
| PA (mg/L) | 58.59±29.93 | 59.25±37.18 | NS |
| ALP(U/L) | 214.74±65.3 | 190.48±61.69 | * |
| UN (mmol/L) | 3.44±1.06 | 3.22±1.11 | NS |
| Cr (umol/L) | 27.49±7.18 | 26.33±5.89 | NS |
| CYSC (mg/L) | 0.81±0.16 | 0.81±0.17 | NS |
| UA (umol/L) | 213.82±79.71 | 207.38±69.16 | NS |
| TC (mmol/L) | 3.28±0.7 | 3.31±0.72 | NS |
| HDLC (mmol/L) | 0.76±0.36 | 0.74±0.32 | NS |
| LDLC (mmol/L) | 3.06±3.65 | 2.86±2.73 | NS |

*<0.05; **<0.01;***<0.001;NS, not significant; BMI, body mass index; N, neutrophil; L, lymphocyte; M, monocyte; RBC, red blood cell; HGB, hemoglobin; PLT, platelet; HCT, Hematocrit; PCT, procalcitonin; CRP, C-reactive protein; γGT, γ glutamyltransferase; PA, serum prealbumin; ALP, alkaline phosphatase; CYSC, Cystatin C.

Continues data were presented as mean ± SD; categorical variables were presented as percentage.
