# Supplementary material for: Alterations in bile acid metabolites associated with pathogenicity and IVIG resistance in Kawasaki disease
Source: Front Cardiovasc Med. 2025 Feb 20;12:1549900. doi: 10.3389/fcvm.2025.1549900 (PMC11882569; doi:10.3389/fcvm.2025.1549900)
Supplement: Supplementary Table S3 — Clinical information of samples of CAL and nCAL. [file Table3.docx]

Table 3. Clinical information of samples of CAL and nCAL

| Variables | nCAL (n=81) | CAL (n=24) | Significance |
| --- | --- | --- | --- |
| Age (years) | 3.09±2.1 | 3.52±3.18 | NS |
| BMI (kg/m^2^) | 16.52±2.61 | 16.95±1.78 | NS |
| **Gender** |  |  | NS |
| Male | 38 | 16 |  |
| Female | 43 | 8 |  |
| **Ethnic** |  |  | NS |
| Minorities | 4 | 0 |  |
| Han Nationality | 77 | 24 |  |
| WBC (×10^9^/L) | 14.4±5.74 | 14.65±7.58 | NS |
| N (%) | 67.96±15.89 | 67.48±16.64 | NS |
| L (%) | 23.56±13.96 | 22.16±11.64 | NS |
| M (%) | 6.15±2.96 | 6.34±3.04 | NS |
| RBC (×10^12^/L) | 4.21±0.51 | 4.15±0.51 | NS |
| HGB (g/L) | 110.46±11.48 | 110.48±10.2 | NS |
| PLT (×10^9^/L) | 356.62±122.04 | 338±122.83 | NS |
| HCT (%) | 33.45±3.27 | 33.98±3.36 | NS |
| PCT (%) | 0.34±0.11 | 0.32±0.09 | NS |
| CRP (mg/L) | 69.71±44.79 | 84.6±49.79 | NS |
| ALT (U/L) | 60.65±78.82 | 64.23±61.78 | NS |
| AST (U/L) | 46.99±46.43 | 46.86±37.42 | NS |
| AST/ALT | 1.41±0.82 | 1.24±0.79 | NS |
| TB (mmol/L) | 11.51±14.61 | 8.62±8.85 | NS |
| DBIL (mmol/L) | 6.27±11.74 | 4.55±7.41 | NS |
| IDIL (mmol/L) | 5.25±3.65 | 3.84±1.66 | NS |
| ALB (g/L) | 40.49±4.52 | 40.76±5.05 | NS |
| GLB (g/L) | 22.12±4.5 | 21.5±3.44 | NS |
| γGT (U/L) | 64.75±84.44 | 63.32±56.78 | NS |
| LDH (U/L) | 316.59±85.87 | 305.5±103.12 | NS |
| PA (mg/L) | 59.09±34.27 | 58.75±37.28 | NS |
| ALP(U/L) | 196.34±64.5 | 208.55±60.93 | NS |
| UN (mmol/L) | 3.28±1.07 | 3.34±1.22 | NS |
| Cr (umol/L) | 27.01±6.03 | 25.73±7.48 | NS |
| CYSC (mg/L) | 0.8±0.18 | 0.84±0.14 | NS |
| UA (umol/L) | 213.36±72.25 | 195±74.21 | NS |
| TC (mmol/L) | 3.36±0.73 | 2.98±0.54 | NS |
| HDLC (mmol/L) | 0.74±0.35 | 0.76±0.29 | NS |
| LDLC (mmol/L) | 3.07±3.32 | 2.22±0.79 | NS |

*<0.05; **<0.01;***<0.001;NS, not significant; BMI, body mass index; N, neutrophil; L, lymphocyte; M, monocyte; RBC, red blood cell; HGB, hemoglobin; PLT, platelet; HCT, Hematocrit; PCT, procalcitonin; CRP, C-reactive protein; γGT, γ glutamyltransferase; PA, serum prealbumin; ALP, alkaline phosphatase; CYSC, Cystatin C.

Continues data were presented as mean ± SD; categorical variables were presented as percentage.
