# Supplementary material for: Alterations in bile acid metabolites associated with pathogenicity and IVIG resistance in Kawasaki disease
Source: Front Cardiovasc Med. 2025 Feb 20;12:1549900. doi: 10.3389/fcvm.2025.1549900 (PMC11882569; doi:10.3389/fcvm.2025.1549900)
Supplement: Supplementary Table S4 — Clinical information of samples of IVIG and rIVIG. [file Table4.docx]

Table 4. Clinical information of samples of IVIG and rIVIG

| Variables | IVIG (n=12) | rIVIG (n=93) | Significance |
| --- | --- | --- | --- |
| Age (years) | 4.25±3.59 | 3.05±2.17 | NS |
| BMI (kg/m^2^) | 15.57±1.98 | 16.75±2.47 | NS |
| **Gender** |  |  | NS |
| Male | 8 | 46 |  |
| Female | 4 | 47 |  |
| **Ethnic** |  |  | NS |
| Minorities | 0 | 4 |  |
| Han Nationality | 12 | 89 |  |
| WBC (×10^9^/L) | 13.99±7.25 | 14.51±6.05 | NS |
| N (%) | 71.73±12.64 | 67.39±16.33 | NS |
| L (%) | 19.7±11.1 | 23.63±13.66 | NS |
| M (%) | 5.7±4.39 | 6.25±2.79 | NS |
| RBC (×10^12^/L) | 4.14±0.42 | 4.2±0.52 | NS |
| HGB (g/L) | 108.45±11.88 | 110.71±11.11 | * |
| PLT (×10^9^/L) | 277.91±97.76 | 361.48±121.82 | NS |
| HCT (%) | 32.72±3.21 | 33.67±3.29 | NS |
| PCT (%) | 0.29±0.11 | 0.34±0.11 | NS |
| CRP (mg/L) | 87.75±53.65 | 71.29±45.16 | NS |
| ALT (U/L) | 61.27±72.44 | 61.44±75.91 | * |
| AST (U/L) | 73.09±106.92 | 43.8±29.24 | NS |
| AST/ALT | 1.44±0.77 | 1.36±0.83 | NS |
| TB (mmol/L) | 10.13±11.79 | 11.01±13.89 | NS |
| DBIL (mmol/L) | 5.75±10.15 | 5.91±11.09 | NS |
| IDIL (mmol/L) | 4.37±1.86 | 5.01±3.51 | NS |
| ALB (g/L) | 38.32±5.99 | 40.82±4.39 | NS |
| GLB (g/L) | 20.21±3.32 | 22.17±4.35 | NS |
| γGT (U/L) | 61.91±62.2 | 64.75±81.12 | NS |
| LDH (U/L) | 352.91±136.07 | 309.52±81.94 | NS |
| PA (mg/L) | 53.82±24.34 | 59.67±35.84 | NS |
| ALP(U/L) | 188±84.35 | 200.16±61.1 | NS |
| UN (mmol/L) | 3.64±1.31 | 3.25±1.07 | NS |
| Cr (umol/L) | 27.55±8.66 | 26.63±6.07 | NS |
| CYSC (mg/L) | 0.86±0.14 | 0.8±0.17 | NS |
| UA (umol/L) | 248.27±74.11 | 204.72±71.4 | NS |
| TC (mmol/L) | 2.83±0.46 | 3.35±0.72 | * |
| HDLC (mmol/L) | 0.5±0.12 | 0.77±0.34 | NS |
| LDLC (mmol/L) | 1.97±0.7 | 3.02±3.19 | NS |

*<0.05; **<0.01;***<0.001;NS, not significant; BMI, body mass index; N, neutrophil; L, lymphocyte; M, monocyte; RBC, red blood cell; HGB, hemoglobin; PLT, platelet; HCT, Hematocrit; PCT, procalcitonin; CRP, C-reactive protein; γGT, γ glutamyltransferase; PA, serum prealbumin; ALP, alkaline phosphatase; CYSC, Cystatin C.

Continues data were presented as mean ± SD; categorical variables were presented as percentage.
